# Aquagenic Wrinkling of the Palm: A Rare Diagnostic Clue of Cystic Fibrosis and the Response to CFTR-Modulating Therapy

**DOI:** 10.7759/cureus.14425

**Published:** 2021-04-11

**Authors:** Paola M Torres-Laboy, Jesus M Melendez-Montañez, Wilfredo De Jesús-Rojas

**Affiliations:** 1 Pediatrics, Ponce Health Sciences University, Ponce, PRI; 2 Biology, University of Puerto Rico, Mayagüez Campus, Mayaguez, PRI; 3 Pediatrics, Ponce Health Sciences University School of Medicine, Ponce, PRI

**Keywords:** aquagenic palmoplantar keratoderma, cystic fibrosis, cftr mutation, genetic screening, skin wrinkling, aquagenic wrinkling of palms, cftr modulating therapy

## Abstract

Aquagenic wrinkling of the palms (AWP), also known as aquagenic palmoplantar keratoderma, is an uncommon dermatosis characterized by transient translucent whitish papules, edema, and hyper-wrinkling of the palms and soles shortly after water immersion. Approximately up to 80% of cases reported are associated with cystic fibrosis (CF) patients and up to 25% with CF carriers. We present the case of a 16-year-old male who complains of new-onset symmetrical edematous wrinkling on his palms associated with brief water exposure. After evaluation and genetic testing, the patient was diagnosed with CF and AWP. While there are numerous theories regarding the pathogenesis of AWP, no consensus has been reached regarding its etiology or relationship with CF. However, given the high prevalence of AWP associated with the genetic disease, physicians should have a high index of suspicion of CF or cystic fibrosis transmembrane regulator (CFTR)-related disease in pediatric patients with this presentation. The presence of AWP as part of the physical examination may help recognize challenging CF cases with uncommon genetic variants. Prompt recognition of CF disease leads to timely initiation of CFTR-modulating therapy, improving the patient’s health outcomes and quality of life. In this case, we also present the patient’s response to CFTR-modulating therapy and compare with baseline status.

## Introduction

Cystic fibrosis (CF) is an autosomal recessive genetic condition in which the c*ystic fibrosis transmembrane regulator* (*CFTR*) gene suffers from one or several mutations disrupting chloride transcellular and paracellular flux in many tissues of the body. Due to the imbalance of electrolytes, individuals suffering from this condition may experience recurrent pulmonary infections, bronchiectasis, pancreatic insufficiency, and failure to thrive [1]. The most common pathogenic mutation leading to systemic disease is c.1521_1523del (deltaF508). However, depending on the type and frequency of mutations, are the organ systems affected and the classification on the CFTR spectrum of disease [1]. *CFTR *mutations also disrupt sweat production on the eccrine glands found in the skin, leading to increased chloride on the epidermis and drawing out sodium and water from the ducts [2]. In 1974, Elliot wrote in a letter to *The Lancet* of immediate palm wrinkling with water exposure present in children with CF. Today, aquagenic wrinkling of the palms (AWP) is seen in up to 80% of CF patients and up to 25% of CF carriers [3].

AWP, or aquagenic palmoplantar keratoderma, is an uncommon condition presenting with transient edema, translucent white papules, or plaques, and wrinkling or thickening of the palms, less often the soles, shortly after water exposure. It often develops bilaterally; however, there have been cases of unilateral presentation [4]. In addition to the cutaneous manifestations, it is frequently associated with pruritus, burning, and pain. Lesions and sensory dysfunction may last from 10 minutes to up to 3 hours upon water exposure termination [4]. The dermatosis usually presents in the second decade of life with equal sex incidence [5]. Although it is a clinical diagnosis, histology shows dilation of eccrine duct ostia with hyperplasia of eccrine glands and acanthosis of the epidermis [2]. The thickness appreciated is due to the uptake of water by the stratum corneum, which can be confused with true hyperkeratosis seen in other diseases, such as hyperhidrosis and hereditary palmoplantar keratoderma [2]. 

Despite the condition being highly prevalent in the CF population, limited information regarding its pathophysiology and association with the autosomal recessive disease is known. However, reports of the condition show that AWP utilized as a screening tool is highly favorable to further explore for CF disease with *CFTR *genetic screening [6]. Individuals with mild disease cases often suffer the most due to delays in treatment. This in effect causes poor health outcomes and a decrease in quality of life. In this case report, we focus on the association of this rare dermatosis with mutations in the CFTR channel. Our goal is to increase awareness of individuals presenting with AWP as a clinical sign for CF or CFTR-related disease (CFTR-RD) for early CF recognition and diagnosis. Furthermore, we present the clinical response of the patient to elexacaftor-tezacaftor-ivacaftor, the most recent combination CFTR-modulating therapy. This case was previously presented as an abstract at the Fourth Interdisciplinary San Juan Bautista School of Medicine Research Symposium on February 20, 2020.

## Case presentation

A 16-year-old Hispanic male with a past medical history of bronchial asthma presented with his caretaker to our institution with complaints of intermittent abdominal pain, wet cough, and poor weight gain since infancy. The patient also had associated foul-smelling, floating stool with inability to gain weight despite a good appetite. He had no history of gastrointestinal disease, multiple respiratory infections, or severe asthma attacks. He also complained of a recent development of symmetric wrinkling with fissures and swelling of his hands’ palmar skin. His caretaker explained noticing the wrinkling during infancy; however, it was not until the start of his new job at a carwash when it became more prominent. Symptoms occurred within 3-5 minutes of water exposure and resolved within minutes after terminating water contact with no residual side effects. He could not associate worsening of wrinkling with water temperature or various bodies of water. There was never itchiness, pain, or burning sensation present with wrinkling. The patient mentioned having dry skin and increased sweating in his palms since infancy but had never been diagnosed with a dermatological condition. He also denied any family member experiencing similar symptoms.

**Figure 1:**
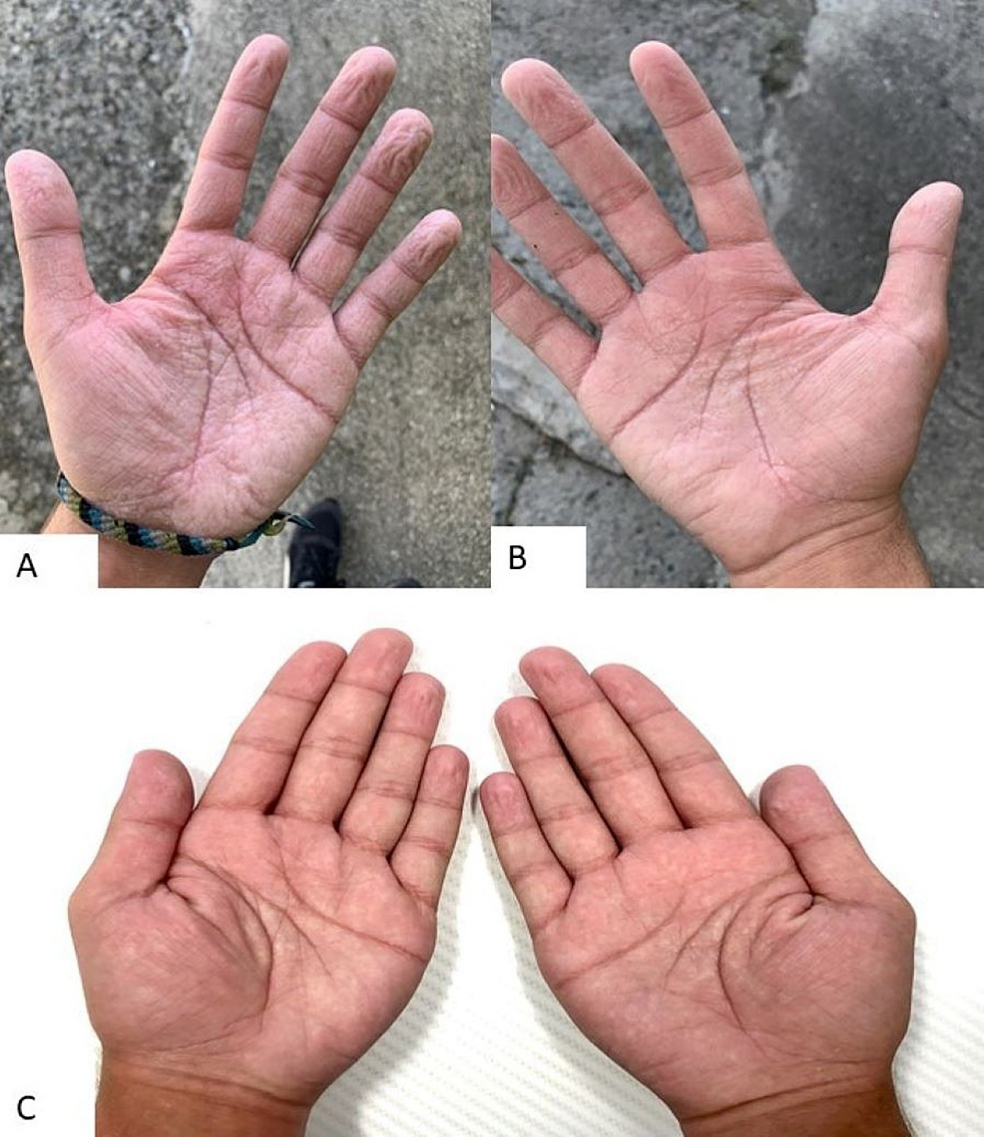
Aquagenic wrinkling of the palms (A, B) Patient’s left and right hands after 5-minute water exposure exhibit symmetric wrinkling with increased fissures and edema on palmar region including interdigital webs but sparing dorsal surface. Findings consistent with aquagenic wrinkling of the palms in a CF patient. (C) Patient’s left and right hands after 5-minute water exposure during his 24-week follow-up since initiating CFTR-modulating therapy with elexacaftor-tezacaftor-ivacaftor. Hands show significant decrease in wrinkling throughout the palmar and interdigital web regions. No fissuring or edema is present. CFTR, cystic fibrosis transmembrane regulator

On the physical examination, the patient’s height and weight were below the third percentile based on age, with his body mass index (BMI) at the 23rd percentile. He also presented with neurodevelopmental delay closer to the age of 11-12 years. Upon mouth inspection, oral mucosa was dry with gingival inflammation and halitosis present. Breath sounds on bilateral lung fields were clear; yet, clubbing was appreciated bilaterally in his upper extremities. The abdomen was mildly distended with normal bowel sounds and non-tender to palpation on all quadrants. The patient was asked to wet his hands on the examination room sink for 5 minutes. Increased thickness and wrinkling with associated blanching and edema was seen on bilateral palms and interdigital webs with sparing of the dorsal surface (Figure 1A, B). No erythema, warmth, or desquamation was appreciated. Based on his history and physical examination findings, a chest high-resolution computerized tomography was ordered, which showed mild bilateral bronchiectasis. However, no acute or infiltrative processes, such as pleural effusion or pneumonia, were appreciated. A pulmonary function test (PFT) was performed following the American Thoracic Society guidelines [7]. Baseline forced vital capacity (FVC) was 71% predicted, forced expired volume in 1 second (FEV1) was 55% predicted, and FEV1/FVC ratio was 78%. Post-bronchodilation with albuterol sulfate demonstrated an FVC of 69% predicted, FEV1 of 58% predicted, and FEV1/FVC ratio of 84% predicted. Additional laboratories demonstrated deficiency in all fat-soluble vitamins (ADEK). A sweat test was performed, exhibiting a concentration of 101 mEq/L (normal range: <60 mEq/L). Subsequently, a sample was sent for CFTR genetic testing, and the patient was diagnosed with two pathogenic heterozygous CF mutations in c.1519_1521del (p.Ile507del) and c.1521_1523 (p.Phe508del). Skin symptoms were attributed to CF-related aquagenic wrinkling of the palms.

The patient was initiated on specialized vitamins (ADEK) and mineral supplements for CF as well as elexacaftor-tezacaftor-ivacaftor (100 mg/50 mg/75 mg). Airway clearance breathing treatments were also prescribed for long-term pulmonary management. As AWP symptoms did not hinder the patient’s quality of life, he elected to use barrier protective methods at work and with water exposure in general. On his 16-week follow-up post elexacaftor-tezacaftor-ivacaftor initiation, PFTs improved to the following baseline FVC of 102% predicted, FEV1 of 102% predicted, and FEV1/FVC ratio of 101% predicted. After 24 weeks of starting treatment with elexacaftor-tezacaftor-ivacaftor, the patient reported reduced gastrointestinal symptoms and increased weight reaching the 55th percentile. Patient also began exercising and reported increased energy levels, exercise time, and endurance. During his follow-up appointment, his hands were submerged in water for 5 minutes with significantly decreased wrinkling and no fissuring present (Figure 1C). The patient mentioned an increase in severity of wrinkling at the beginning of treatment with gradual improvement. He was encouraged to continue with his current treatment with close follow-up to monitor for improvement or adverse effects from medication. 

## Discussion

We presented the case of newly diagnosed compound heterozygous CF with associated AWP in an adolescent male. Pediatric patients with atypical symptoms of CF, like in our patient, may go undiagnosed until adolescence or early adulthood. This delay in recognition causes detrimental physical and neurocognitive delays. Most AWP cases reported have been predominantly associated with CF; however, the condition's pathophysiology and its connection with CF is not yet understood. It was thought to be primarily due to CFTR channel dysfunction and high salt concentration on the epidermis creating an osmotic gradient and increasing the water-binding capacity [3]. Nonetheless, in recent population studies, there has been no relationship between the sweat chloride concentration in CF patients and the severity of AWP [1,8,9]. The CFTR protein is also known to regulate the expression of aquaporin (AQP) channels in epithelia. CFTR dysfunction might lead to an increased expression of AQP channels as seen in clinically similar diseases, such as hereditary palmoplantar keratoderma, Bohnia type, as well as hyperhidrosis [10]. A 2012 prospective observational study measuring transepidermal water loss (TEWL) favored this hypothesis. In CF patients with AWP, the mean value of TEWL was 282.4 g/m^2^/h, CF with no AWP was 209.7g/m^2^/h, and the control group was 173.1 g/m^2^/h. In normal skin physiology, TEWL should decrease once the wrinkling due to water retention is present. This finding is consistent with increased expression of AQP in the surface, letting more water into the tissue [3].

Although AWP is predominantly described in CF patients, there have been cases of medication-induced, acquired cases in healthy individuals and in patients with marasmus, atopic dermatitis, and hyperhidrosis [4,9]. Medication-induced AWP has been reported with cyclooxygenase 2 inhibitors, non-steroidal anti-inflammatory drugs, gabapentin, acetylcholinesterase enzyme inhibitors, aldosterone receptor blockers, and the aminoglycoside antibiotic tobramycin [9]. These medications are known to affect and potentiate sodium retention or the osmotic gradient within epithelia. It is important to note that most of the cases reported did not test the individual for CFTR genotype mutations, and one cannot dismiss the possibility of the medication causing a synergistic effect with an already present condition [11]. Other hypotheses studied are genetic pathways closely linked to CFTR expression, epidermal barrier or eccrine duct dysfunction, and abnormal sympathetic stimulation of the nerve fibers, among others [11]. Multiple studies have attempted to find a specific genotype within the CFTR protein that may further provide evidence to the pathophysiology of AWP. Nevertheless, no consistent pattern relating CFTR genotype and AWP development has been established [8].

From the earlier cases reported, it was thought that AWP was only present in homozygous individuals with the deltaF508 mutation. However, in 2010, Gild et al. published a case report describing the manifestation in both homozygous and heterozygous CF variants [2,12]. Patients presenting with AWP and normal sweat chloride tests were sent for expanded CFTR genetic testing and were found to have uncommon CFTR variant mutations. Because a sweat chloride test alone cannot completely exclude CF, normal sweat chloride test should not deter a physician from sending a patient for CFTR genetic screening and consider CF as a diagnosis [3,13]. About 5% of patients have uncommon pathogenic CF variants, as in our patient. This presents with fewer organ systems affected, leading to delayed recognition or complete miss of the condition altogether. The widely used CFTR screening panel tests for the 23 most common mutations are as recommended by the American College of Obstetrics and Gynecology and American College of Medical Genetics. With over 2,000 CFTR mutations and a high false-negative rate screening panel, numerous uncommon pathogenic variants may be missed [14,15]. Furthermore, this genetic panel does not identify CF carriers who also suffer from milder disease forms. If one concludes evaluation with the standard CFTR clinical screening tool, diagnosis of such a debilitating disease may be missed. AWP may provide a clue to patients who might need expanded genetic screening to further evaluate for CFTR spectrum of disease and manage possible life-altering conditions [10].

One obstacle to this proposal is the lack of standardized parameters or grading for AWP [2,5,9]. With different time intervals in which symptomatology appears in CF and CF carriers, there is difficulty stratifying patients in studies to understand the condition further. Additionally, there is no set percentage of surface area to diagnose AWP, leaving physicians uncertain in milder presentations of the disease [4]. Underreporting of the condition presents yet another obstacle to the wide use of AWP as a screening tool. Many patients with CF, who suffer from severe chronic health conditions, may find this complaint as insignificant to report during appointments [5]. If a patient presents with pancreatic insufficiency, abdominal discomfort, respiratory disease, or has failure to thrive, physicians should ask about the cutaneous manifestation to further guide the evaluation of possible CFTR-RD and provide more data on the usefulness of AWP as a screening tool [12]. Hence, further research is needed to create a global assessment scale to standardize asking for AWP symptomatology in patients with atypical CF presentation.

Early diagnosis of CF provides considerable nutritional, cognitive, and physical survival benefits compared to late CF diagnosis. Treatment is dependent on the affliction presented by the patient. Typically, supplementation of fat-soluble vitamins (ADEK) and pancreatic enzymes, and electrolyte correction improve growth and development, preventing stunted physical and neurodevelopmental delay. Newer CFTR-modulating therapies are also an option in patients with specific gene variants targeting the underlying disease itself. Once our patient’s genotype was known, he was initiated on elexacaftor-tezacaftor-ivacaftor (100 mg/50 mg/75 mg). Consisting of potentiators and correctors, it is only available for individuals with at least one deltaF508del mutation present, allowing most of the affected individuals to benefit from gene-modulating therapy. After 24 weeks on elexacaftor-tezacaftor-ivacaftor medication, our patient had a significant decrease in clinical symptomatology with an improvement in BMI, as expected [15].

During his follow-up appointment at 24 weeks, the hand-water immersion test showed a significant decrease in AWP wrinkling (Figure 1C). This observation favors the correlation between CF and AWP, linking a dysfunction in the CFTR protein to the cutaneous manifestation. As combination therapy not only aids in the transporting of the CFTR protein channel to the surface but also increasing its stability to facilitate ion transport, normal functioning of the CFTR protein corrects any downstream dysregulation created [16]. Comparison of AQP expression with initiation of combination therapy may provide answers to the pathogenesis of AWP. A change in electrolyte concentration and AQP expression may explain the worsening symptoms the patient experienced after initiating treatment with gradual improvement to what was observed. Additional reports regarding this observation must be studied in order to further make this association. Treatments currently used for AWP include topical 5-20% aluminum hydroxide or botulinum toxin injections, among others [11]. In the adolescent male case presented, the patient was using water barrier options with a gradual improvement of symptoms after initiation of CFTR modulating therapy. Although AWP was not a bothersome symptom to this patient, reporting it during his appointment provided an immense clue in diagnosing the actual cause of his other debilitating symptoms.

## Conclusions

Pediatric patients with atypical CF presentation may go undiagnosed until adolescence or early adulthood, causing detrimental health and neurocognitive conditions and increasing their morbidity and mortality. Identification of this rare dermatosis may present as the only sign of an underlying mutation within the *CFTR *gene. With the numerous genetic mutations present within *CFTR *and the variable symptomatology that could present, sweat chloride testing may be normal and halt any further evaluation for CFTR-RD. For this reason, physicians must have a low threshold to unmask CF via expanded *CFTR *genetic screening. Before utilizing AWP as a screening tool, further research needs to be done to learn about its etiology, association with CF, and standardization of parameters.

It is hoped that greater awareness of the prevalence of AWP in the CF population will lead to a better understanding of the condition's pathophysiology. Patients suffering from other symptomatology might not disclose wrinkling of their palms during appointments, believing it to be insignificant or normal. Thus, asking about the cutaneous manifestation should be part of the initial interview with any patient presenting with atypical symptoms. As CFTR-modulating therapy becomes more readily available, systemic improvement can be observed, such as decreased severity of AWP. Studying the effects of this therapy could also clarify the pathogenesis of the disease and improve related symptomatology. However, a decrease in the cutaneous manifestations with the medication provides evidence of the possible association between the two rare conditions. AWP may also be used as a qualitative guide to the improvement of systemic disease with modulating therapy. As more therapies are being studied to expand the genotype pool able to use them, we may see a clearer association of CFTR correction and its effects in AWP.
